# Role of exosomes in non-small cell lung cancer and EGFR-mutated lung cancer

**DOI:** 10.3389/fimmu.2023.1142539

**Published:** 2023-04-12

**Authors:** Ding-Yu Rao, De-Fa Huang, Mao-Yan Si, Hua Lu, Zhi-Xian Tang, Zu-Xiong Zhang

**Affiliations:** ^1^Department of Cardiothoracic Surgery, The First Affiliated Hospital of Gannan Medical University, Ganzhou, China; ^2^Laboratory Medicine, The First Affiliated Hospital of Gannan Medical University, Ganzhou, China; ^3^The First Clinical College, Gannan Medical University, Ganzhou, China; ^4^The First Clinical College, Southern Medical University, Guangzhou, China

**Keywords:** exosome, EGFR, lung cancer, target therapy, tumor microenvironment (TME), epithelial to mesenchymal transformation (EMT)

## Abstract

As an important mediator of information transfer between cells, exosomes play a unique role in regulating tumor growth, supporting vascular proliferation, tumor invasion, and metastasis. Exosomes are widely present in various body fluids, and therefore they can be used as a potential tool for non-invasive liquid biopsy. The present study reviews the role of exosomes in liquid biopsy, tumor microenvironment formation, and epithelial-mesenchymal transition in non-small cell lung cancer (NSCLC). By targeting epidermal growth factor receptor (EGFR) therapy as a first-line treatment for patients with NSCLC, this study also briefly describes the occurrence of EGRF+ exosomes and the role of exosomes and their contents in non-invasive detection and potential therapeutic targets in EGFR-mutated lung cancer.

## Introduction

Lung cancer is the main malignant tumor leading to human death in the world today (1). According to the data of World Health Organization GLOBOCAN 2020, the statistical data on the incidence and mortality of 36 kinds of cancers in 185 countries show that: Although the incidence of female breast cancer (11.7%) is higher than that of lung cancer (11.4%), lung cancer is still the leading cause of cancer mortality (18%), with about 1.8 million people dying from lung cancer every year (2). The five-year survival rate of early lung cancer can reach 56%. Since early lung cancer has no obvious clinical symptoms, when patients have clinical symptoms, the stage is often late. Most patients are found in the middle and late stage, and only about 16% of patients can be found in the early stage (3). It is morphologically classified into two main subtypes: small cell lung cancer (SCLC), which accounts for approximately 15% of all lung cancers, and non-small cell lung cancer (NSCLC), accounting for approximately 85%. The three major subtypes of NSCLC are adenocarcinoma (~50% of all NSCLC), squamous cell carcinoma (~30%), and large cell undifferentiated carcinoma (~15%) (4). Smoking is the leading risk factor for lung cancer. Other risk factors for lung cancer include a poor diet, genetic changes, occupational exposure, and air pollution (5). The availability of high-resolution computed tomography and advances in immunotherapy and molecularly targeted therapies have significantly improved the current state of lung cancer treatment. However, the prognosis of NSCLC remains poor, with a five-year survival rate of 10% (6).

It is crucial to master the biological mechanisms inherent to tumor development and identify potential cancer biomarkers for early diagnosis, targeted therapy, and drug development. Exosomes are secreted by cells with small extracellular vesicles (sEVs) with double membrane structures. Almost all cells can secrete exosomes; however, tumor cells often secrete more exosomes than normal cells. Exosomes are distributed in various human body fluids, including blood plasma, saliva, breast milk, cerebrospinal fluid, etc. (7, 8). Exosomes are membrane nanovesicles released from extracellular luminal vesicles after the fusion of multivesicular bodies with the plasma membrane. Recipient cells can take up exosomes emitted by donor cells in an autocrine, paracrine, or endocrine manner, thus demonstrating the critical role of exosomes in intercellular communication (9). Transfer of functional exosomal content to recipient cells can lead to pathological or physiological effects (10). An increasing number of studies are being conducted on the progression of exosomes as novel mediators of intercellular communication in multiple types of cancers, including lung carcinogenesis and the tumor microenvironment (TME). Thus, exosomes are known for mediating intercellular communication during tumor development (11–14). The current review provides an overview of the role of exosomes in NSCLC growth, metastasis, and immune response. In addition, the potential role of exosomes as non-invasive biomarkers in EGFR-mutated NSCLC is discussed.

## Effect of exoPD-L1 in NSCLC

PD-L1, also known as cluster of differentiation CD274 or B7 homolog B7-h1, is a type I transmembrane protein encoded by the CD274 gene (15). In 1992, Tasuku Honjo and his colleagues at the University of Tokyo identified PD-1 as a membrane protein expressed on T cells associated with apoptosis and suggested that the PD-1 product might have its own ligand (16, 17). Later, it was found that PD-L1 is widely expressed not only on T cells but also on a variety of cells, mainly on tumor cells, macrophages, monocytes, natural killer (NK) cells, dendritic cells (DCs), but also in immune-specific sites such as the brain, cornea and retina (18). Study found that in human blood, PD-L1 exists in three main forms, one is expressed on the plasma membrane PD-L1, the other is expressed on the surface of secreted cell exosomes, or as circulating soluble PD-L1 (sPD-L1).

Physiologically, the PD-1/PD-L1 pathway emerged because of the need to control the degree of inflammation at the site of antigen expression in order to protect normal tissues from damage. Almost all activated T cells significantly express PD-1 protein on their surface (19). When T cells recognize the Major Histocompatibility Complex (MHC) antigen on target cells, inflammatory cytokines are produced to initiate the process of inflammation. Some cytokines cause tissue cells to express PD-L1, which inhibits T cell activation and leads to immune tolerance, causing the immune system to lose control over the initiation of the inflammatory response even in the presence of a viable antigen (20). In some tumors, most notably in melanoma, this protective mechanism is disrupted by overexpression of PD-L1. PD-1/PD-L1 inhibitors pharmacologically block the PD-1/PD-L1 interaction, thereby promoting an aggressive immune response to kill the tumor. It was reported that compared to renal cell carcinoma and melanoma, the expression level of PD-L1 in NSCLC levels were significantly higher (21). High expression levels of PD-L1 were positively correlated with Progression-Free Survival (PFS) and Overall Survival (OS) after treatment with PD-1/PD-L1 inhibitors (22). Study (21) found that in human blood, PD-L1 exists in three main forms, one is expressed on the plasma membrane PD-L1, the other is expressed on the surface of secreted cell exosomes (exoPD-L1), or as circulating soluble PD-L1 (sPD-L1).

Tumor cell PD-L1 is clinically recognized as a predictor of response to immunotherapy. PD-L1 is also the immune-related biomarker of lung cancer with the highest level recommended by the current guidelines and is the most widely used predictive marker in clinical application. Although PD-L1 in tumor tissue is an indicator authorized by United States Food and Drug Administration (FDA), the expression pattern of PD-L1 on tumor cells alone is not sufficient to accurately predict tumor response to anti-PD-1/PD-L1 therapy. The use of membrane PD-L1 has drawbacks such as invasive biopsy, heterogeneity of PD-L1 expression within tumors, inability to perform dynamic observations, and limited sensitivity (21). Studies have shown that, Circulating exoPD-L1 is emerging as a non-invasive and readily available biomarker and is a more readily detectable and reliable surrogate than sPD-L1 in tissue and plasma (23, 24).

Yang et al. (25) collected paired tissue samples and blood samples from 51 patients with advanced NSCLC to detect the dynamic changes of PD-L1 expression in blood of patients with advanced NSCLC after 2 months of Immune Checkpoint Inhibitors (ICIs) treatment, including the changes of PD-L1 mRNA, exoPD-L1 protein and sPD-L1. Among 40 patients with advanced NSCLC, patients with ≥2.04 fold change in PD-L1 mRNA had better PFS, OS, and best overall response (BOR). In addition, in a group of 21 patients with advanced NSCLC, a fold change of ≥1.86 for exoPD-L1 was found to be associated with better efficacy and OS, whereas the dynamics of sPD-L1 were not. This suggests that increased PD-L1 mRNA and/or exoPD-L1 expression in the early phase of ICIs treatment may serve as a positive biomarker for efficacy and OS in patients with advanced NSCLC. In addition, the combination of PD-L1 mRNA and exoPD-L1 may allow better screening of patients for the potential benefit of ICIs treatment. Further studies (25, 26) suggested that miR-21 contained in PD-L1-positive exosomes may be a biomarker to differentiate between NSCLC patients and healthy controls. Yang et al. (27) detected the expression of miR-21 containing EGFR or PD-L1 exosomes and thyroid transcription factor-1 (TTF-1) mRNA in human serum with immunochip, and obtained the absolute sensitivity and specificity for distinguishing normal controls from NSCLC patients. In A549 EGFR-positive exosomes, miR-21 and TTF-1 mRNA levels were 1.6-and 2.8-fold higher, respectively, than in BEAS-2B cells. Meanwhile, PD-L1-positive exosomes from A549 cells had 5.3-and 5.9-fold higher miR-21 and TTF-1 mRNA levels, respectively, than BEAS-2B cells. These results suggest that PD-L1-positive exosomes miR-21 and TTF-1 mRNA have better tumor recognition performance than EGFR-positive exosomes. This suggests that EGFR-positive and PD-L1-positive exosomes miR-21 and TTF-1 mRNA are effective serum biomarkers to differentiate NSCLC patients from healthy controls.

In addition, Ricklefs et al. (28) screened 85 patients with newly diagnosed NSCLC and 27 healthy subjects as the study subjects, and analyzed the correlation between the immunohistochemical characteristics of exoPD-L1, sPD-L1 and PD-L1 and the clinicopathological characteristics. The results showed higher levels of exoPD-L1 in patients with NSCLC, especially in advanced stages, compared to healthy controls. In addition, in NSCLC patients, high exoPD-L1 content was associated with tumor size, positive lymph node status, distant metastasis, and tumor-node-metastasis (TNM) stage. However, there was no significant difference in serum sPD-L1 level between NSCLC patients and healthy subjects, and there was no correlation between serum sPD-L1 level and other clinicopathological features except tumor size (> 2.5 cm) (P> 0.05). In summary, exoPD-L1 was associated with NSCLC disease progression, including tumor size, nodal status, metastasis, and TNM staging.

Those studies suggest that exoPD-L1 may be an effective biomarker for the diagnosis and treatment of lung cancer. However, it should be noted that the utility of exoPD-L1 as a diagnostic biomarker needs to be further validated in large patient cohorts, in patients with other lung cancer histologies (e.g. squamous NSCLC, small cell lung cancer), and in patients with early stages of disease (e.g. stage I).

## Role of exosomes in NSCLC liquid biopsy

The high mortality rate of lung cancer patients is mainly due to the late diagnosis of the disease (29). Patients are usually admitted with symptoms of chest tightness, hemoptysis, or systemic involvement. The 5-year survival rate for lung cancer patients with distant metastases is as low as 4% (30), and the overall 5-year survival rate is only 24% (31). Therefore, elucidating the mechanism underlying lung cancer metastasis is of great significance to identify relevant biomarkers for early diagnosis and precise treatment in patients with lung cancer.

“Liquid biopsy” is a non-invasive or minimally invasive disease detection method based on molecular diagnostic techniques (32). This technique has become a hot research topic in recent years. It is different from the traditional surgical biopsy technique and puncture biopsy technique, and mainly uses the body fluids of cancer patients such as blood, urine, breast milk, and saliva to detect circulating biomarkers of tumors, and to obtain relevant genetic information of the disease (33). It provides new ideas and methods for the early diagnosis and treatment of diseases. Liquid biopsy has numerous advantages, including easy to use, non-invasive (34), low cost, low side effects (35), easy sample collection, repeatable sampling, less harm to patients, and high level of patient acceptability (36). It enables early detection of tumors than imaging techniques and is suitable for the early diagnosis of related diseases. It also provides a novel approach for early diagnosis and adjuvant clinical treatment of lung cancer patients, which can improve the prognosis and the quality of life and reduce the mortality rate of lung cancer patients.

**Figure 1:**
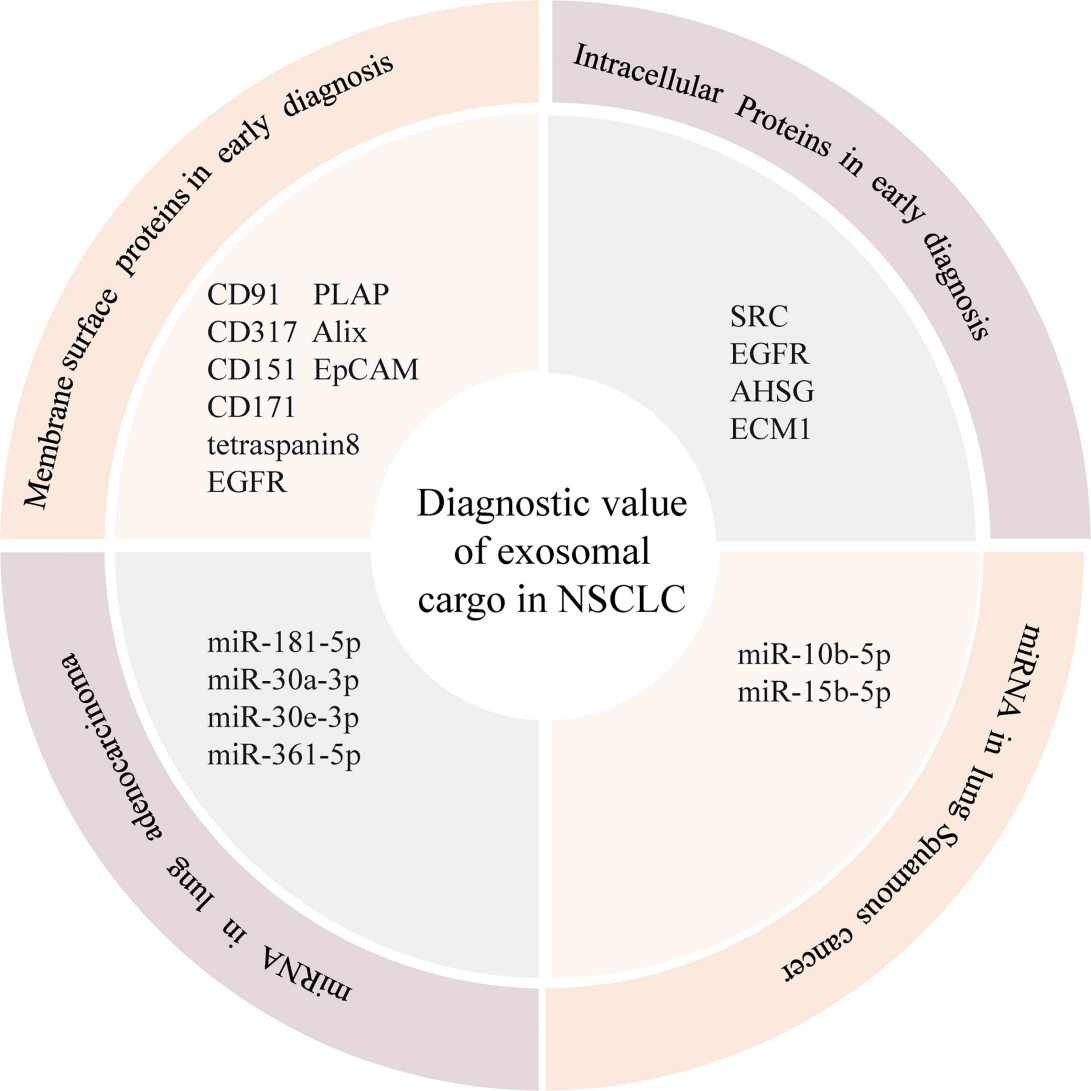
Diagnostic value of exosomal cargo in NSCLC.

The use of exosomes as biomarkers in NSCLC is a promising approach in the era of liquid biopsy. Several studies have recently shown that exosomes are more stable and their contents have greater similarity to parental cells because they are present in most body fluids. Genetic material and information related to the parental cells can be obtained by detecting and extracting the contents of the exosomes (37). Exosomes in various body fluids are abundant, specific, and uniform in size, and contain abundant tumor-related genetic information such as specific biomarkers (e.g., RNAs, DNAs, lipids, and proteins) and other biomolecules, which directly or indirectly regulate the expression of recipient cells and play an important regulatory role in the development of tumors (see **Figure 1**). A previous study found that the exosomal membrane surface protein NY-ESO-1 from the plasma of patients with lung cancer was significantly associated with poorer survival (38).

Exosomes secreted by tumors can be effectively detected in almost all types of body fluids. The composition of exosomes is extremely complex, containing many proteins, nucleic acids, etc., and varies greatly among diseases, individuals, and even disease stages. Exosomes contain microRNAs (miRNAs), proteins, and other biomolecules that are more stable than free miRNAs and proteins, and can accurately reflect the physiological state and pathological characteristics of secretory cells. As a marker for liquid biopsy, exosomes have good biological prospects.

## Exosomal protein markers

Exosomes contain various protein components such as surface and intracellular proteins, which can contribute to the development of lung cancer and are closely related to the early diagnosis and prognosis of lung cancer (39). So far, multiple signature exosomal membrane proteins — such as CD91, CD317 — can be used as diagnostic biomarkers for lung cancer (40). Research shows that the use of a mixture of biotin-conjugated CD151, CD171, and tetraspanin8 antibodies to detect and capture exosomes has a greater ability to isolate exosomes than traditional exosome extraction methods (38). More importantly, the levels of exosomal CD151, CD171, and tetraspanin8 were found to be highly expressed in lung cancer patients, and their expression levels were significantly higher than those in normal subjects, suggesting that CD151, CD171, and tetraspanin8 are potentially potent protein markers for early diagnosis of lung cancer. In addition, exosomal membrane surface proteins such as EGFR, placental alkaline phosphatase, epithelial cell adhesion molecule (EpCAM), and Alix are significant predictors of long-term overall survival in lung cancer patients and can be used as potential prognostic markers (41). It is worth mentioning that Koji et al. found that the expression of CD91 in the exosome was significantly increased, especially in the serum of patients with lung adenocarcinoma, and suggested that the exosome with high level of serum cd91 expression might initially be secreted by stromal cells around lung cancer cells (40, 42). CD91 may be the lung adenocarcinoma specific antigen on the exosome. In addition to the surface signature exosomal membrane proteins, it has been found that exosomal proteins can also be used as biomarkers for early diagnosis of lung cancer. David et al. (43) analyzed 721 exosomal proteins using a quantitative proteomic approach and identified several proliferation-related cell signaling molecules, including SRC, EGFR, and other signal transduction-related proteins, which are enriched in NSCLC exosomes and can positively regulate tumor recipient cell proliferation. The study of the NSCLC exosome proteome has helped to identify exosome-enriched protein substances associated with lung cancer progression, which may have potential clinical implications for the discovery and development of NSCLC biomarkers. Niu et al. (44) explored tumor-derived exosomal biomarkers in the sera of 125 NSCLC patients and 46 normal subjects to improve the diagnostic value of NSCLC patients. The expression levels of alpha-2-HS-glycoprotein (AHSG) and extracellular matrix protein 1 (ECM1) in the exosomes of NSCLC patients were significantly higher than those of the healthy controls, indicating that AHSG and ECM1 in serum exosomes of NSCLC patients have a potential diagnostic value.

## Exosomal nucleic acid markers

Exosomal RNA is an important component of its inclusions, and the expression level of exosomal RNA in lung cancer patients is significantly higher than that of the normal population, which is closely related to the biological characteristics of lung cancer such as development, invasion, and metastasis (45). In recent years, a large body of literature has reported that circulating exosomal miRNAs can be used as potential diagnostic markers for lung cancer. Cazzoli et al. (46) identified four miRNAs (miR-378a, miR-379, miR-139-5p, and miR-200b-5p) from plasma exosomes of 30 subjects to screen and distinguish lung cancer patients from healthy controls. Similar to exosomal proteins, the expression level of exosomal miRNA can also be used as an indicator to assess the prognosis of lung cancer patients. Liu et al. (47) showed that high expression levels of exosomal miRNAs (miR-23b3p, miR-10b-5p, and miR-21-5p) were significantly associated with poor prognosis in lung cancer patients, and the combined analysis of multiple miRNAs had higher sensitivity and specificity. Cecilia et al. (48) found that increased expression of miR-21 was associated with a worse prognosis in NSCLC patients. Among the miRNAs of NSCLC-derived exosomes, miR-10b-5p and miR-15b-5p were found to be specific for the diagnosis of squamous carcinoma, while miR-181-5p, miR-30a-3p, miR-30e-3p, and miR-361-5p were specific for the diagnosis of adenocarcinoma (49). According to Zhang et al. (32), the expression of exosomal miR500a-3p, miR-501-3p, and miR-502-3p was significantly upregulated in lung cancer patients after surgery (50), suggesting that the three miRNAs may be associated with tumor progression and these changes may also be associated with a persistent inflammatory response during tumor growth. Further analysis revealed that the upregulation of miR-500a-3p, miR-501-3p, and miR-502-3p was associated with improved overall survival in lung cancer patients, and although the mechanisms underlying the occurrence of the three miRNAs in lung cancer are unknown, their tumor-promoting effects have been observed in other cancer types. This suggests that exosomal miR-500a-3p, miR-501-3p, and miR-502-3p have great potential as early diagnostic markers for lung cancer. Therefore, the detection of differentially expressed exosomal miRNAs using a non-invasive method (liquid biopsy) can be used for the early diagnosis of lung cancer, providing a new technical consideration and a solid theoretical basis for the early diagnosis of lung cancer.

## Role of exosomes in the TME of NSCLC

**Figure 2:**
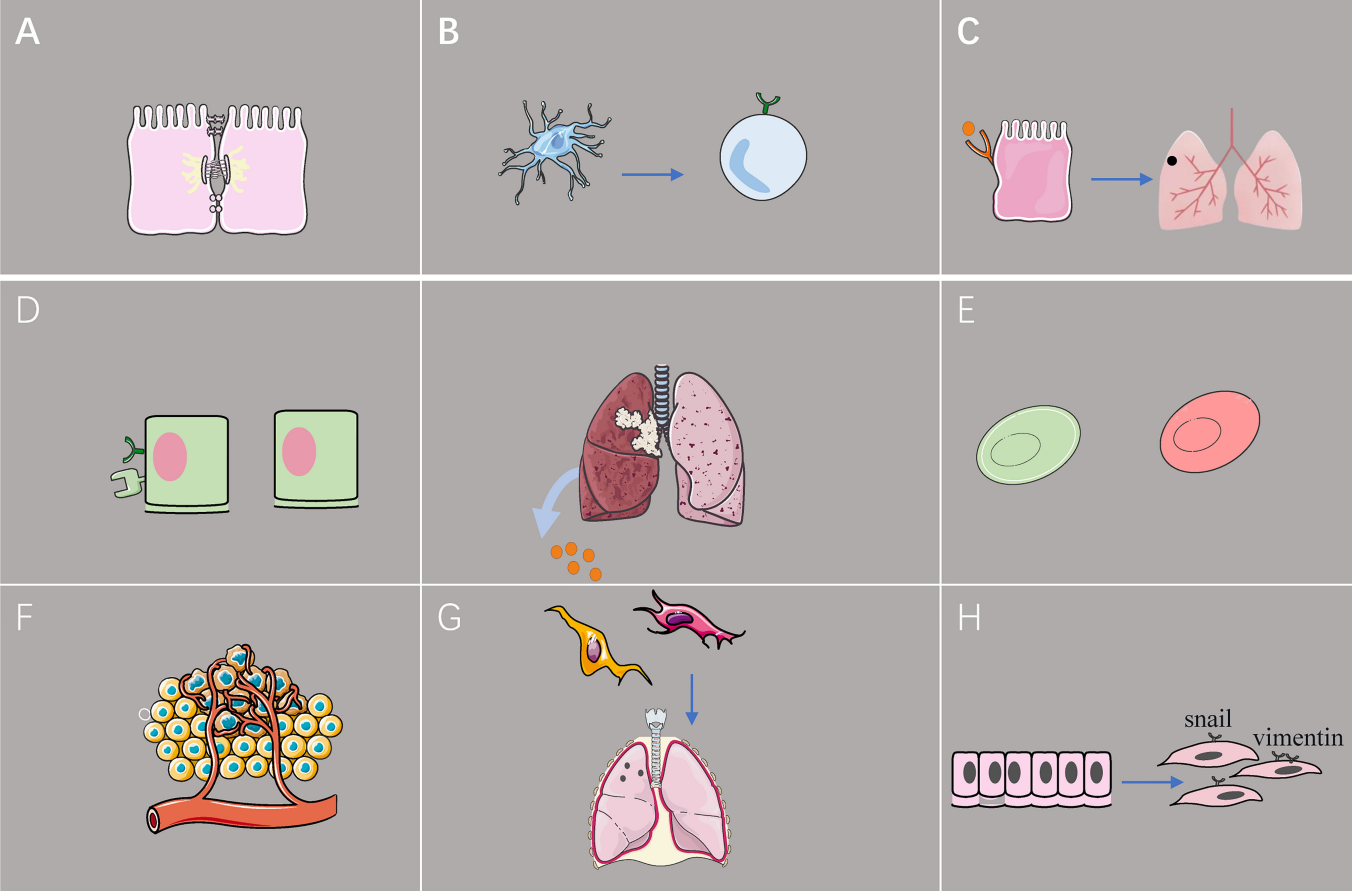
The effect of exosome from lung cancer cell on TME. **(A)** Exosomal miR-23a inhibits the tight junction protein ZO-1, increases vascular permeability, and promotes cancer cell migration. **(B)** Tumor-derived exosomes reduce programmed death-ligand 1 expression in dendritic cells, leading to downregulation of the population of regulatory T cells *in vitro*. **(C)** Lung epithelial cells sense tumor exosomal RNA *via* Toll-like receptor 3 and promote the formation of pulmonary pre-metastatic niches. **(D)** Hypoxic lung cancer cells promote M2 macrophage polarization by secreting excessive amounts of exosomal miR-21. **(E)** Exosomes induce early glycolytic reactions, thereby lowering the pH of the tumor microenvironment and promoting macrophage activation. **(F)** Upregulation of exosomal miR-210 promotes human umbilical vein endothelial cells and leads to increased tumor angiogenesis. **(G)** Exosomes promote the conversion of fibroblasts to tumor-associated fibroblasts, and exosomes from tumor-associated fibroblasts induce the formation of pre-metastatic niches in the lungs of mice. **(H)** Exosomes acquired from the sera of patients with advanced lung cancer have increased expression of vimentin and trigger epithelial-to-mesenchymal transition of lung cancer cells.

Exosomes have long been thought of as waste products of cellular metabolism. Accumulating studies have demonstrated that exosomes are increasingly important in cellular communication (10, 51, 52), especially in regulating tumor growth, supporting vascular proliferation, tumor invasion, and metastasis (53, 54). Exosomes can promote the formation of the TME (see **Figure 2**) (55). TME consists of the tumor vasculature, extracellular matrix (ECM), and other supporting cells such as stromal cells, fibroblasts, and inflammatory cells (56). Therefore, therapeutic strategies targeting the TME may be a promising approach for cancer (57–59).

NSCLC releases a large number of extracellular vesicles, mainly in the form of exosomes (60–62), Exosomes regulate immune mechanisms by regulating antigen presentation, immune activation, immune suppression, immune surveillance, and intercellular communication (63–65). Exosome-stimulated-DCs of lung cancer cell-associated antigens activate CD4+ T and CD8+ T lymphocytes to induce antitumor immune responses (66). CD40 ligand-modified lung cancer cell exosomes effectively activate DCs, inhibit lung cancer progression, and prolong survival in mice (67). Rab-27a high expression of non-small cell lung cancer cell exosomes also effectively stimulated the proliferation and maturation of DCs, which subsequently significantly increased the proliferation of CD4+ T cells, acting as an immunomodulatory agent (68). DC vaccine-based immunotherapy is emerging as a new cancer treatment strategy, however, the anti-tumor efficacy of DC vaccines based on tumor cell lysates (TCL) remains unsatisfactory due to the poor immunogenicity of tumor antigens. Recently, Wang et al. found that tumor-derived exosomes more effectively promoted DC maturation and enhanced MHC cross-presentation, which directly contributed to a more potent tumor-specific cytotoxic T lymphocyte (CTL) response. More importantly, exosomes reduced PD-L1 expression in DCs, leading to downregulation of the Tregs population *in vitro*, and significantly inhibited lung cancer cell growth and prolonged survival *in vivo* (69). Thus, tumor-derived exosomes trigger stronger DC-mediated immune responses and reduce Treg in the tumor microenvironment.

Hypoxia, a common phenomenon in the tumor microenvironment, can alter tumor metabolism and thus affect cancer progression (70). Thus, cells cultured under hypoxic conditions can mimic the *in vivo* environment. There is growing evidence that hypoxia promotes cancer development by promoting exosomes secreted by cancer cells (71). Exosomal miR-23a targets prolyl hydroxylases 1 and 2 (PHD1 and 2) and inhibits endothelial cell expression. Hypoxia-inducible factor-1α (HIF-1α) accumulates in endothelial cells, thereby increasing angiogenesis. In addition, exosomal miR-23a inhibits the tight junction protein ZO-1, which is associated with vascular permeability and cancer cell migration (72).

Tumor-associated macrophages (TAM) are one of the immune cell populations of TME that usually lead to poor prognosis in patients with malignancies (73). Macrophages are thought to be divided into two major classes, classically activated macrophages (M1) and selectively activated macrophages (M2).M1 macrophages exhibit mainly pro-inflammatory activity, secreting a variety of pro-inflammatory factors such as tumor necrosis factor (TNF), nitric oxide, interleukin 1 (IL-1) and interleukin 12 (IL-12). On the other hand, M2 macrophages exhibit potent anti-inflammatory activity, countering the pro-inflammatory response of M1 by upregulating IL-10 and downregulating IL-12 with markers including CD206, CD163 and CD68 (74). Previously, it was reported that macrophage M2 polarization is generated by the tumor microenvironment, and then polarized M2 macrophages promote tumor cell proliferation, invasion, and EMT (75–77). Researchers found that hypoxia induced tumor-associated macrophage enrichment and M2 polarization *via* HIF. Downregulation of HIFs expression inhibits glioma progression by reducing M2 polarization and TAM infiltration (78), It was recently found that hypoxic lung cancer cells promote macrophage M2 polarization by secreting excessive amounts of the exosome miR-21. miR-21 targets the 3′ UTR of interferon regulatory factor 1 (IRF1) and downregulates macrophage IRF1 expression. Polarized M2 macrophages further promote lung cancer cell proliferation (79). In addition to non-coding RNAs, researchers found that hypoxic lung cancer cells secrete large amounts of exosomal PKM3, which mediates the AMPK/p38 pathway to induce M2 polarization in macrophages, thereby promoting tumor progression (80).

Unlike normal cells, the metabolism of tumor cells may rely primarily on glycolysis, and even under aerobic conditions, these cells may produce relatively high amounts of lactate, thereby lowering the pH of TME (81). Macrophage activation significantly increases glycolysis-dependent ATP production, while exosomes induce early glycolytic responses and promote macrophage activation (82–84).

Exosomes can act not only on macrophages but also on other cells in the TME, such as immune cells, endothelial cells, fibroblasts and other cells that disrupt the host immune system and drive tumor progression (85–87). Tumor cells producing p53 mutations secrete exosomes that mediate functional intercellular metastasis by increasing Rab protein-dependent integrin recycling in other tumor cells (88, 89). Tissue inhibitor matrix metalloproteinase-1 (both exogenous and endogenous) leads to the upregulation of exosomal miR-210 in the CD63/phosphoinositide 3-kinases (PI3K)/serine/threonine kinase (AKT)/HIF-1-dependent pathway in lung adenocarcinoma cells, promotes tube-forming activity in human umbilical vein endothelial cells, and leads to increased tumor angiogenesis (90).

Tumor-associated fibroblasts (CAFs) are the most predominant component of tumor stromal cells in the TME. CAFs are in extensive contact with tumor cells and can affect other components of the TME (91–93). Contrary to normal fibroblasts, CAF can secrete a variety of growth factors, cytokines, and ECM. These factors play a crucial role in promoting tumorigenesis, proliferation, tumor angiogenesis, invasion, and metastasis (94–96). It was previously found that miR-210, an exosome secreted by lung cancer cells, acts on fibroblasts. The ten-11 translocation 2 and JAK2/STAT3 signaling pathways of CAFs are targets during angiogenesis, which promote the release of the angiogenic factors vascular endothelial growth factor (VEGF), matrix metalloproteinase-9, and fibroblast growth factor 2 (72). Exosomes overexpressing miR-210 can activate the function of CAFs and increase the expression of pro-angiogenic factors (97). Overexpression of tissue inhibitor of metalloproteinases-1 also leads to the accumulation of miR-210 in exosomes, which promotes angiogenesis (98). CAFs also deliver the transcription factor Snail homolog 1 (SNAI1) to lung cancer cells *via* exosomes and induce EMT through cadherin-1 encoding epithelial-cadherin and vimentin (VIM) encoding waveform proteins (99). In addition, exosome-associated miR-142-3P promotes the conversion of lung fibroblasts to CAFs through transforming growth factor-β (TGF-β) signaling (100).

Natural killer cells (NK) are independent, non-specific immune cells. They can kill tumor cells directly without MHC restriction to the target (83, 101). However, tumor cells interfere with the normal function of NK cells and attenuate the cytotoxicity of TME. The degree of NK cell infiltration was positively correlated with the survival rate in lung cancer patients (102). Recent studies have shown that exosomes from NK cells also have antitumor properties. In addition, these NK cell-derived exosomes have higher stability, greater modification potential and less immunogenicity than NK cells (103). Researchers isolated NK cells and circulating tumor cells (CTCs) from a small cohort of patients with NSCLC and found that NSCLC patients had higher numbers of NK and NK-exosomes compared with healthy donors, and that these concentrations tended to correlate positively and negatively, respectively, with the number of blood-borne CTCs. It was further demonstrated that NK-exosomes obtained from NK-cells had cytotoxic effects on CTCs (103). DNAX accessory molecule-1 (DNAM1), is a key receptor of NK cells. DNAM1 is expressed more in infiltrating NK cells of primary lung tumors compared to the expression in surrounding normal tissues. In recent study, Researchers found NK cells have a cytolytic effect in lung tumors *via* exosomal DNAM1 receptor-ligand binding and endocytosis (104).

## Role of exosomes in NSCLC metastasis

Tumor metastasis is a complex process that involves the transformation of quiescent epithelial cells into motile cells and the invasion of other organs, a phenomenon known as EMT. EMT is a process in which polarized epithelial cells (mostly interacting with the basement membrane through their basal surface) undergo a variety of biochemical changes that result in a mesenchymal cell phenotype, including increased migratory and invasive capacity, increased resistance to apoptosis, and increased production of ECM components (105–107). EMT is signaled by the degradation of the underlying basement membrane and the formation of mesenchymal cells, allowing migration of mesenchymal cells from their origin to the upper cortex. EMT weakens the strong adhesion between differentiated epithelial cells, allowing cancer cells to achieve individual or collective motility, which makes EMT an intuitive mechanism for tumor metastasis initiation (107–109). Exosomes can be involved in the EMT effect of tumor cells (110–112).

EMT is regulated by TGF-β (113). Previous studies demonstrated that human umbilical cord mesenchymal stem cells (MSCs) promoted EMT, invasion, and migration of A549 lung cancer cells *via* MSC-derived exosomes (MSC-Exos). Inhibition of TGF-β1 expression reversed the EMT-promoting effect and enhanced the anti-growth effect of MSC-Exos on lung cancer cells. MSC-Exo promotes EMT in A549 cells *via* mothers against decapentaplegic homolog 2/3 (SMAD2/3), AKT/glycogen synthase kinase-3β, mitogen-activated protein kinase, and nuclear factor kappa B pathways. The inhibition of TGF-β1 expression in MSC inactivates SMAD-dependent and non-dependent pathways activated by MSC-Exos (114).

EMT transcription factors (EMT-TFs, including Snail1, Snail2/Slug, etc.) contribute significantly to the development of EMT, and EMT-TFs can induce the expression of mesenchymal genes (e.g., VIM, fibronectin 1, N-calcineurin (CDH2)); therefore, Snail and VIM can be considered as mesenchymal-specific markers. Exosomes derived from transplanted lung cancer cells induce the expression of VIM and EMT in human bronchial epithelial cells. In addition, it was shown that exosomes obtained from the sera of patients with advanced lung cancer had increased expression of VIM and induced a more metastatic phenotype in recipient cells, suggesting that exosomes can trigger EMT of lung cancer cells (37).

Exosome-mediated transfer of miRNAs (including miR-193a-3p, miR-210-3p, and miR-5100) from bone marrow mesenchymal stem cells to epithelial cancer cells activates signal transducer and activator of transcription 3 (STAT3) signaling and increases the expression of mesenchymal-associated molecules (Snail and VIM), induces EMT, and promotes invasion of lung cancer cells (115).

Lung cancer cells produced more exosomes under oxygen deprivation conditions, where exosomal miR-23a is significantly upregulated. This led to the accumulation of HIF-1α in endothelial cells, which promoted angiogenesis and permeability, as well as tumor migration (72).

Exosomes from the sera of EGFR-mutant NSCLC patients mediate the activation of mammalian targets of the PI3K/AKT/rapamycin (mTOR) pathway and induce invasion through upregulation of matrix metalloproteinase-9 in A549 cells. The expression of VIM was significantly increased and epithelial features such as epithelial-cadherin and EpCAM levels were unchanged. Moreover, the expression of nuclear factor red lineage 2-related factor 2 and placental (P)-cadherin increased, which are markers of mixed EMT. Thus, exosomes from EGFR-mutant adenocarcinoma sera may be potential mediators of mixed EMT and tumor invasion (64).

Tumor-secreted exosomes can also promote the formation of pre-metastatic niches (PMNs). Exosomes secreted by tumor cells form PMNs at distant metastatic sites (107, 116). The stromal environment of PMNs consists mainly of fibroblasts, endothelial cells, and ECM. Fibroblasts not only induce inflammation and growth factors but also express fibronectin and matrix metalloproteinases (117), promoting the degradation of the ECM. Tumor-associated fibroblast-derived exosomes induced the formation of PMNs in the lungs of mice and increased lung metastasis of salivary gland cystic carcinoma (118). Liu et al. (119) showed that lung epithelial cells sense tumor exosomal RNA *via* Toll-like receptor 3, which is essential for initiating neutrophil recruitment and formation of lung PMNs, providing the right conditions for tumor metastasis.

## Role of exosomes in EGFR-mutated lung cancer

The understanding of cancer genomic alterations has enabled the identification of potential diagnostic and therapeutic targets, one of which is EGFR. EGFR mutations are predominant in lung adenocarcinoma, ranging from 10% to 78%, and varying significantly by race and geographic location (120, 121). Recent findings suggest that cancers with EGFR mutations are associated with an increased incidence of diffuse lung metastases (122). The development of EGFR tyrosine kinase inhibitors (EGFR-TKIs) has revolutionized the treatment of lung cancer. EGFR-TKIs are now recognized as the first-line treatment of NSCLC patients harboring EGFR mutations (L858R missense mutations in exon 19 and exon 21) (123).

## Diagnostic value of EGFR^+^ exosomes

Current guidelines strongly recommend molecular testing for screening lung cancer patients (124). However, tissue biopsy is either inconclusive or unavailable in 20% of patients due to a lack of sufficient tumor tissue or because it is not technically feasible (125). Thus, liquid biopsy is the potential complementary/alternative tool to conventional tissue biopsy for diagnosis and prognosis (126). Exosomes can transport tumor molecules (DNA and RNA), and the results of exosome nucleic acid analysis suggest that exosomes are sensitive in identifying relevant mutations (43, 127). Exosome-derived EGFR may be a differential marker for diagnosing NSCLC and chronic inflammatory lung disease. Previous studies found that approximately 80% of exosomes obtained from NSCLC biopsies were EGFR-positive, compared with only 2% of those from chronic inflammatory lung tissue (128). Current studies on non-invasive tumor markers focus on circulating tumor cells, circulating tumor DNA (CtDNA), circulating free DNA (CfDNA), and other relevant biomarkers. So far, the detection of EGFR is mainly based on nucleic acids of ctDNA origin, which are currently being applied in clinical practice (125). Cobas EGFR Mutation Test V2^®^ (Roche Diagnostics Inc.) was the first liquid biopsy test to be approved by the United States Food and Drug Administration (FDA) (129). This test allows the analysis of mutations present in cfDNA fragments, such as exon 19 deletions or exon 21 (L858R) substitution mutations in the EGFR gene. However, its ability to detect EGFR-T790M has only 58% sensitivity and 80% specificity. Thus, even by using the most sensitive analytical platform, the nature of ctDNA and the methodological limitations of detection complicate the liquid biopsy of cfDNA (130, 131). To address these issues, recent studies have revealed the advantages of combining identified exosomal nucleic acid (exoNA) mutations with cfDNA for mutation detection (132–134). Moreover, Fernando et al. (135) suggested that 93% of plasma cfDNA was localized to exosomes. The value of exosomal RNA in identifying tumor-derived somatic mutations has also been proven. A previous study included a parallel screening of exosomal RNA and cfDNA (stage IIIB, IV) from 84 EGFR-positive NSCLC patients. It was found that the sensitivity for detection of activating EGFR mutations and EGFR-T790M was 98% and 90% for exosomal RNA and 82% and 84% for CFDNA, respectively (134). Exosomal RNA can be used as a biomarker for EGFR mutations in lung cancer. However, the sample sizes of the above studies were relatively small, and therefore these findings should be validated using a larger cohort.

## Occurrence of EGFR^+^ exosome

**Figure 3:**
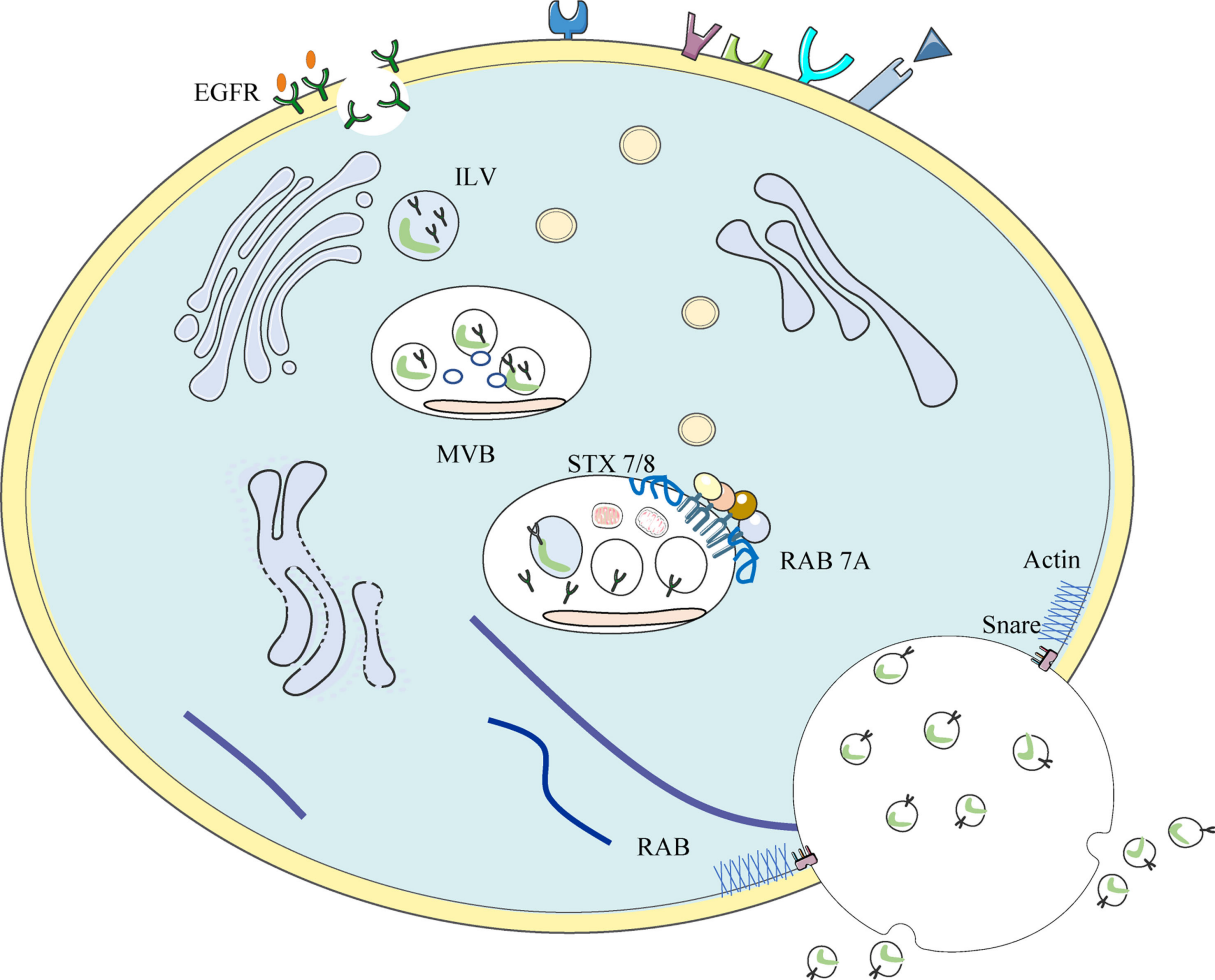
Occurrence of EGFR^+^ exosome: Invaginations of the cell membrane containing activated EGFR form membrane bound vacuoles known as early endosomes. Following a series of changes, endosomes mature into late endosomes and subsequently, *via* the inward budding of the endosomal membrane, membrane-enclosed vesicles called intra-luminal vesicles (ILVs) are formed within the endosome. Multiple inward budding events fill endosomes with ILVs; at this stage endosomes become referred to as multivesicular bodies (MVBs).

EGFR-loaded exosomes are formed during EGFR endolysosomal transport (see **Figure 3**) (136). Membrane-bound vacuoles formed by the invagination of cell membranes containing activated EGFR is called early endosome (137). After a series of changes, endosomes mature into late endosomes, followed by the formation of membrane-enclosed vesicles called intraluminal vesicles (ILVs) within the endosomes by inward outgrowth of the endosomal membrane. ILVs are the earliest stage of exosomes. Multiple inward outgrowth events fill the intranucleosome with ILV; at this stage, the intranucleosome is called the multivesicular body (MVB). The MVB showing specific surface proteins (including EGFR) binds to lysosomes, leading to the degradation of ILV contents. Other proteins shown on the MVB include the GTPase RAS-associated protein RAB 7A, the HSP 70-HSP 90 histone protein (HOP) complex, and members of the membrane fusion soluble N-ethylmaleimide-sensitive factor attachment protein receptor (SNARE) complex, including vesicle-associated membrane protein 7 (VAMP 7) and synaptic fusion proteins 7 and 8 (STX 7/8), which label the MVB for lysosomal degradation (138–140). MVBS required for exosome formation are translocated along microtubules to the plasma membrane. Rabs, actin, and SNARE proteins mediate the fusion of MVBS with the cell membrane and the subsequent release of ILVs into the extracellular space. At this point, ILVs are referred to as exosomes (141).

## EGFR^+^ exosomes as therapeutic targets

EGFR is involved in the biogenesis of specific extracellular vesicle subpopulations. It signals as an active cargo and influences the uptake of exosomes by receptor cells. EGFR regulates its inclusion in exosomes during disease progression through a feedback loop and in response to hypoxia (142), EMT (64, 143), medications, etc. (136, 144). EGFR, its oncogenic mutants, and its signaling network proteins are commonly expressed in lung cancer exosomes of different origins, which leads to the activation of translational signaling pathways and regulation of target gene expression, such as VEGF, anti-apoptotic B-cell lymphoma-extra-large, and cell cyclin-dependent kinase inhibitor P27 (145–147). It has been previously demonstrated that EGFR can transfer to endothelial cells *via* exosomes, activating mitogen-activated protein kinase and AKT pathways and promoting VEGF expression, thereby increasing the expression of anti-apoptotic genes and non-anchored growth capacity (148). In addition to promoting angiogenesis, EGFR-containing lung cancer exosomes can also translocate to host macrophages, thereby suppressing intrinsic antiviral immunity and enabling immune escape (149). In addition to acting on macrophages, Exo-EGFR inhibits the tumor immune function of DCs (150).

Resistance to EGFR-TKIs is also associated with exosomal contents. Liu et al. (151) found that T790M-mutant cell exosomes induce resistance to gefitinib in sensitive cells through activation of the PI3K/Akt signaling pathway. Exosome transfer of wild-type EGFR also confers resistance to osimertinib by activating the PI3K/Akt pathway (152). However, Chen et al. (146) demonstrated that exosomal miRNAs can play a role in reversing gefitinib resistance. They found that exosomal miR-7 is resistant to gefitinib by promoting phosphorylation of YAP, an effector of the HIPPO pathway. Collectively, these studies suggest a mechanism of resistance of NSCLC to EGFR-TKIs *via* exosomes.

## Summary

As liquid biopsy takes the center stage as a tool for the diagnosis and management of cancer, extracellular vesicles are increasingly recognized as an attractive method to obtain non-genetic molecular information about solid tumors through minimally invasive approaches (153). In this context, the identification of subclasses of extracellular vesicles derived from cancer cells and microenvironmental components associated with well-defined pathological processes would allow them to serve as complementary biomarkers of circulating cfDNA and circulating tumor cells. In recent years, several studies have demonstrated the value of exosomes as cancer biomarkers, allowing longitudinal monitoring of tumor heterogeneity and early identification of cancer subtypes (154, 155), as well as monitoring microenvironmental subversion (156), tumor progression and prognostic decisions (157), and response to therapy to tailor therapeutic interventions (158, 159). Further developments in this field will bring us closer to the most important goal of providing personalized cancer care. In terms of clinical applications, multiple non-coding RNAs and proteins have been identified in exosomes, which may be indispensable tools for the diagnosis and prediction of lung cancer in the clinical setting. However, there is a lack of clinical studies with large samples to provide evidence to support this. It is particularly important to identify the precise components that play a key role in tumor pathogenesis.

Current techniques for exosome extraction and isolation fail to meet the requirements for highly sensitive extraction and isolation of exosomes needed for liquid biopsies. EGFR and its signaling network proteins are widely present in the exosomes of patients with NSCLC and are potential biomarkers in oncology research and clinical applications. Accelerating the discovery, validation, regulatory approval, and ultimately rapid use of exosome biomarkers in clinical practice is crucial to the rational development of medical therapeutics. ExoNA measurements may expand the utility of exosomes as a potential diagnostic and prognostic tool for EGFR-mutated cancers, as they may provide a more complete assessment of tumor progression and response to targeted therapies. This may provide new scientific avenues for the development of novel technologies for accurate early detection and diagnosis, staging, precise treatment, and prognosis of lung cancer.

## Author contributions

D-YR and D-FH searched for literature and wrote the first draft of this article. M-YS edited tables and figures. Z-XT and Z-XZ strictly reviewed the manuscript and polished the grammar. All authors approved the final version submitted and agree on its submission to this journal.
